# Current Knowledge on Interspecific Hybrid Palm Oils as Food and Food Ingredient

**DOI:** 10.3390/foods9050631

**Published:** 2020-05-14

**Authors:** Massimo Mozzon, Roberta Foligni, Cinzia Mannozzi

**Affiliations:** Department of Agricultural, Food and Environmental Sciences, Università Politecnica delle Marche, Via Brecce Bianche 10, 60131 Ancona, Italy; m.mozzon@staff.univpm.it

**Keywords:** interspecific hybrid palm, palm oil, ripening, *Elaeis oleifera*, tocotrienols, positional analysis, fatty acids, triacylglycerols, refining

## Abstract

The consumers’ opinion concerning conventional palm (*Elaeis guineensis*) oil is negatively affected by environmental and nutritional issues. However, oils extracted from drupes of interspecific hybrids *Elaeis oleifera* × *E. guineensis* are getting more and more interest, due to their chemical and nutritional properties. Unsaturated fatty acids (oleic and linoleic) are the most abundant constituents (60%–80% of total fatty acids) of hybrid palm oil (HPO) and are mainly acylated in position sn-2 of the glycerol backbone. Carotenes and tocotrienols are the most interesting components of the unsaponifiable matter, even if their amount in crude oils varies greatly. The Codex Committee on Fats and Oils recently provided HPO the “dignity” of codified fat substance for human consumption and defined the physical and chemical parameters for genuine crude oils. However, only few researches have been conducted to date on the functional and technological properties of HPO, thus limiting its utilization in food industry. Recent studies on the nutritional effects of HPO softened the initial enthusiasm about the “tropical equivalent of olive oil”, suggesting that the overconsumption of HPO in the most-consumed processed foods should be carefully monitored.

## 1. Introduction

Palm oil (PO) is obtained from the reddish mesocarp of the African palm (*Elaeis guineensis* Jacq.) (*Eg*) drupes and, to a much lesser extent, of the South American palm (*Elaeis oleifera* (H.B.K.) Cortés) (*Eo*) fruits. The hard woody endocarp encloses the kernel, which provides the palm kernel oil (PKO). Palm drupes are classified into *dura* type (thick-shelled with a thin mesocarp; high oil content), *pisifera* type (shell-less, with a thick mesocarp containing only a small amount of oil), and *tenera* type (thin-shelled, with an abundant pulp and high oil content). The last one is a cross between *dura* and *pisifera* and is currently the kind of fruit from all commercial African palm varieties, whereas the morphology of the *E. oleifera* drupes resemble *dura* fruit type [1,2]. The African oil palm is profitably cultivated in Asian, African, and American tropical belt, whereas the American oil palm spontaneously grows from the south of Mexico to Brazilian and Colombian Amazon areas.

Palm oil is the most produced and marketed vegetable oil all over the world (75.70 million metric tons in the marketing year 2019/2020), due to its high productivity along with the perennial nature of plants, thus resulting in the production of a low-cost oil. Malaysia and Indonesia are both the main palm oil producers, globally accounting for about 85% of the world production, and exporters, followed by Thailand, Colombia, and Nigeria. In addition, the global production of palm oil is still expected to grow in parallel with the population growth in the emerging markets, the increasing demand of trans-free fats and oils as well as the utilization for biofuel production [3].

Food uses of PO and PKO are very diversified as frying and cooking baths (crisps, French fries), margarine and spreads, ingredients for baked foods, confectionary, dairy replacements, prepared foods (salads, sandwiches, pizza, peanut butter, quiche, dressing, sauces, snacks), and fat supplements in pet foods. PO and PKO can also be fractionated and chemically modified into palm-derived food additives, such as colors, antioxidants, emulsifiers, stabilizers, and thickeners. The wide range of applications of palm oils are due to their fatty acid (FA) composition, which results in high oxidative stability, structural hardness, and slow crystallization properties [4]. The quality of palm oil in deep-fat frying has already been proven [5]. Saturated fatty acids (SFAs) and unsaturated fatty acids (UFAs) are almost equally present in PO; with palmitic acid (P) being the most represented SFA (80%–90% of SFA) and oleic acid (O) accounting for 75%–80% of UFA; whereas, lauric acid (La, about 45% of total FAs) and SFA (about 80% of total FAs) predominate in PKO [6,7,8]. In comparison, ‘O’ varies within 50%–70%, linoleic acid (L) ranges from 10%–20%, and ‘P’ accounts only for 15%–20% of total SFA in the oil of *Eo* fruits [9,10].

Despite their functional and technological properties, the reputation of palm oils is getting worse among a wide range of consumers, especially in some European countries (France, Italy, and Spain), in parallel with increasing health and environmental awareness. In fact, the relationship between an SFA-rich diet and risk of coronary heart diseases is now well established in large amounts of people in developed countries. It is also a fact that the massive demand for palm oil has resulted in an increase of extensive monocultures. However, the ecological impact of this expansion depends on the extent to which it is the real cause of deforestation (and of related problems, such as reduction of natural habitats for some animal species like Sumatran tigers, rhinoceros, Asian elephants, Sumatran and Bornean orangutans, and peatland destruction) [11], and on the extent to which it supports biodiversity [12].

Nowadays, the interspecific crossbreeding between the cultivated (African) palm oil and its wild (American) relative (O × G) is gaining increasing attention by agronomists and researchers. The American oil palm has a significantly lower oil production than *Eg*, but it has some interesting traits that are inherited by hybrids, such as better oil quality, due to higher UFA percentage, longer productive life, shorter trunk, and resistance to the main diseases affecting palms [2]. Mesocarp oil content of *Eg* drupes ranges from 75% to 82% dry matter (DM), while *Eo* fruits exhibit a significantly lower and wider range of total lipid content (17% to 60% DM), possibly related to their wider genetic diversity than *Eg* varieties [13]. Hybrid palm fruits have intermediate but still considerable oil content [9,14,15]. Unlike the broad variation found in *Eo* mesocarp, *Eo* kernel lipids only range from 37% to 43% DM; whereas, higher oil levels characterize *Eg* kernels (45%–51% DM) and the kernel oil contents of interspecific hybrids (38%–52% DM) are close to those of their African parent. [8,16] The oil content of hybrids quickly increases between 18 and 24 weeks after anthesis (WAA) [17].

The need to develop varieties resistant to diseases, especially to the “bud rot” [18], has been the main thrust in the study of oil palm breeding, since the pioneering work of Hardon and co-workers [14,19]. In the last years, the devastating spread of the bud rot disease in Central and South America is giving a new boost to the implementation of molecular breeding programs, in order to improve both the oil production traits (fruit morphology, bunch number and weight, oil yield) and the oil quality traits (triacylglycerol composition, tocols and carotenoids content) of hybrids [2,20,21,22,23,24,25]. In fact, O × G hybrids represent the only economically feasible alternative in those regions of Central and South America where the bud rot disease is lethal [26,27,28].

However, several factors are limiting the commercial use of hybrids: lack of seed production corresponding to the best hybrids [29]; uneven filling and ripening because of the asynchronous flower bud opening [17]; lack of data to determine the optimal harvest time to ensure both high oil yield and quality [30]. It has also been reported that hybrid oil palms release a lower amount of pollen with a lower viability than African palm [31,32], thus reducing their attractivity for insects. Therefore, human assisted pollination is mandatory to obtain a high oil production, to the detriment of production costs and crop management. Recently, Alves Filho et al. [33] studied the volatiles produced by male and female inflorescence of different oil palm species (African, Amazonian, and their hybrid) in order to better understand the relationship between plant and pollinators. The inefficiency of procedures to induce the seed germination and the conventional propagation method was also highlighted [34,35,36,37].

Recent papers describe several characteristics of O × G hybrids, such as phenological stages, bunch morphology and yield [24,38,39,40,41,42,43], agronomic performance [44,45,46,47], fruit abscission process [48], and genome size [15,49,50]. Even the mycorrhization process [51] and hydroponic cultivation [52] have been assessed, but limited investigations have been carried out regarding the interspecific hybrid palm oil (HPO) composition and its use in food products.

Therefore, the unique composition of HPO (fatty acid and glyceride composition and structure, unsaponifiable matter constituents), its changes during fruit ripening, and the available data concerning the technological and nutritional properties of HPO are the scope of this review.

## 2. Chemical Composition of HPO

### 2.1. Fatty Acids

Palmitic and oleic acids globally account for about 80% of total FAs (Table 1). 

The O/P (oleic/palmitic acid) ratio significantly differs among HPO (1.5–1.9), PO (around 1.0), and *Eo* oil (lower than 1). As expected, the FA composition of F1 hybrids lies between the FA fingerprints of their African and American parents [6,8], whereas the FA distribution in the F2 generation (F1 × F1) resembles the composition of F1. This behavior has been attributed to a codominant and additive heredity in hybrid palms [55]. Gas chromatographic analysis revealed the presence of small amounts (0.7%–1%) of Δ11 isomer (*cis*-vaccenic acid), together with oleic acid (C18:1Δ9). Some minor FAs (C8:0, C10:0, C15:0, C17:0, C20:0, C:22:0, C24:0, C22:1Δ13, C24:1Δ15) were also identified, which in total represent less than 1% of total FAs [6].

The optimal harvest time of fruit bunches is of paramount importance for oil yield and quality, but only few researches have been focused on chemical changes of hybrid palm fruits during ripening. Lucci et al. [30] observed a decrease of ‘P’ (from 40.3% to 32.2%) and stearic (S) acid (from 3.8% to 2.7%), and a corresponding increase of ‘O’ (from 49.7% to 57.0%) and linoleic (L) acid (from 4.1% to 5.6%) in cold pressed oils from hybrid palm drupes collected 18–24 WAA. Conversely, other researchers reported an increase of ‘P’ (from 28.1% to 31.3%) and a decrease of ‘O’ (from 56.4% to 51.8%), but in a different ripening period (24–27 WAA) [17].

In plants, the de novo synthesis of FAs and the triacylglycerol (TAG) assembly occur in separate compartments. FA biosynthesis takes place in the plastid stroma through the action of the fatty acid synthase complex (FAS); acetyl-CoA carboxylase provides malonyl-CoA needed for elongation of the growing FA chain, which is held by an acyl carrier protein (ACP). The final products are the SFAs C16:0 and C18:0, which can be further subjected to other modifications (elongation, desaturation). Different condensing enzymes (β-ketoacyl-ACP synthase, KAS) characterized by different specificity are used in plants for controlling the final ratio of C16/C18 products. The stearoyl/palmitoyl-ACP Δ9-desaturase is also present in chloroplast stroma and converts C16:0/C18:0 to C16:1/C18:1. Subsequently, acyl-ACPs are hydrolyzed by acyl-ACP thioesterases and the non-esterified FAs exported to the endoplasmic reticulum for TAG assembly, through sequential acylation of the glycerol backbone. Glycerol-3-phosphate acyltransferase (GPAT), lyso-phosphatidic acid acyltransferase (LPAAT), and diacylglycerol acyltransferase (DGAT) are the key condensing enzymes. Acyl-CoA synthetase re-esterifies FAs to CoA after export from plastid [57]. Several studies reported the relationships between the hereditariness and expression of genes encoding the key enzymes Δ9-desaturase and KAS II (which is specifically involved in chain lengthening of ’P’ to ’S’) and the FA composition of HPOs [1,10,58,59,60]. Interestingly, the percentage of linoleic acid in HPO does not significantly deviate from the content of this essential FA in African palm oil. In this case, *Eg* seems to be dominant, while ‘P’ and ‘O’ show additive or co-dominance effects [6,8,55,56].

While great attention was paid to the oil extracted from mesocarp, less emphasis has been reserved to kernel oil from *Eg*, *Eo*, and their hybrids (Table 2). Unlike the mesocarp oil, the kernel oil composition of interspecific hybrids is close to the FA composition of the *Eg* relative. Medium chain FAs, lauric (La) and myristic (M), globally account for more than 60% of total FAs, while ‘O’ percentage ranges from 13% to 19%. Only traces of *cis*-vaccenic acid (amounts lower than 0.1%) have been detected in O × G interspecific hybrid palm kernel oil [8,9,16,61].

### 2.2. Acylglycerols

#### 2.2.1. Triacylglycerols

TAG structure has important nutritional implications because it affects the bioavailability of FAs. The sn-1,3 regiospecificity of human pancreatic lipase induces preferential absorption of FA in the sn-2 position, as 2-monoacylglycerols (2-MAGs). The incorporation of free FAs into mixed micelles is variable, depending on the chain length and degree of unsaturation [62].

Information about the structure and composition of TAG molecular species can be obtained by the combination of mass spectrometry and powerful chromatographic separations, both in liquid [63,64] and gas phase [6]. Mozzon et al. [6,30] identified 23 different TAG types (Table 3), by direct gas chromatography–mass spectrometry analysis of Colombian HPO samples. A preparative thin layer chromatography (TLC) allowed the same authors to identify trisaturated (LaLa8:0, LaLa10:0, LaLaLa, LaLaM, LaMM + LaLaP, LaMP, LaPP + MMP) and disaturated (LaLaO, LaMO) TAG species characterized by one to three medium-chain SFAs (from 8:0 to 14:0). About 70% of medium chain TAGs have three (LaLaLa) or two (LaLaM, LaLaP, LaLaO) lauryl groups.

From a qualitative viewpoint, no differences between the TAG species of HPO and conventional PO have been observed. TAG quantitation of HPO samples shows a predominance (about 80% of total TAGs) of disaturated (mainly PPO) and monosaturated (mainly POO and PLO) TAG types. HPO shows higher relative percentages of monosaturated (47.5%–51.0% vs. 36.7%–37.1%) and triunsaturated TAGs (15.5%–15.6% vs. 5.2%–5.4%) than oil of African palm. It is interesting to notice that hybridization causes a reduction of arachidic acid (A) level but an increase of TAG types (SOA and AOO) characterized by the presence of ‘A’ [6]. Other studies carried out on HPOs of similar FA composition showed different TAG profiles, which were characterized by lower percentages of PPO and POO and higher amounts of PPL, PLO, OOL, and PLL + POLn [15,17]. According to the total number of acyl carbon atoms (CN), the TAG distribution of palm oils obtained from fruit pulp shows a typical unimodal distribution; the apex ranges from CN value of 50–52 (African palm oils) to 52–54 (*Eo* oils), while HPO has the intermediate value (52) [6,9]. It has been highlighted that there is an apparent higher tendency of the hybrid palm to make C52 TAGs (mainly POO, PLO) than those of olive and oleaginous seeds, despite their total FA compositions. This could be a limiting factor in achieving the unsaturation level observed for olive oil and the major seed oils [9].

**Figure 1:**
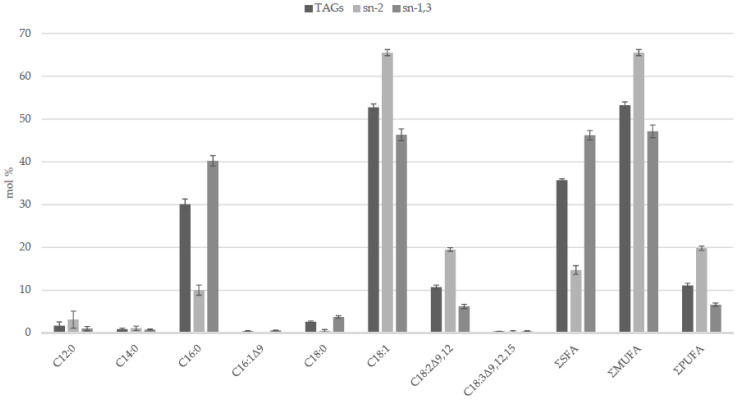
Regiospecific distribution of fatty acids in TAGs of O × G hybrid palm oil. Data from [6]. Fatty acid formula is: C (number of carbon atoms):n (number of double bonds) Δx (position of double bonds). C18:1 = sum of oleic and *cis*-vaccenic acids. SFAs = saturated fatty acids. MUFAs = monounsaturated fatty acids. PUFAs = polyunsaturated fatty acids.

Both enzymatic and chemical procedures were developed for elucidating the FA distribution on the glycerol backbone of complex lipids [64,65,66]: the term “regiospecific” refers to analytical procedures aimed to study the FAs esterified in the sn-2 position separately from those esterified to the primary hydroxy groups of glycerol; the term “stereospecific” refers to procedures that are able to determine the FA profiles esterified to each of the three positions of the glycerol backbone. Experimental data revealed a preferential esterification of SFAs (palmitic and stearic) in the sn-1,3 positions (mol% of FA in sn-1,3 higher than in TAGs), while unsaturated fatty acids (oleic and linoleic) are mainly acylated in position sn-2 (mol % of FA in sn-2 higher than in TAGs) (Figure 1). The asymmetric structure of HPO TAGs suggests that the chain length and the unsaturation degree of FAs could be the discriminating factors in the selectivity of the acylating enzymes [6,55]. It seems that interspecific crossbreeding does not affect the TAG structure, because of the closeness of the FA regiodistribution in TAGs of HPO and oils of its African parent [6].

Very few data are available about the evolution of TAG types during the ripening of hybrid palm drupes. Lucci et al. [30] observed a strong decrease of total trisaturated TAGs, which halved from 3.6% to 1.8%, and an increase in the total amount of triunsaturated TAGs, from 16.9%  to 18.9 %, mainly due to the increase of OOO and the decrease of PPP.

#### 2.2.2. Partial Glycerides

Edible fats and oils mainly consist of TAGs, but small amounts of partial glycerides (diacylglycerols, DAGs; monoacylglycerols, MAGs) are always present, as intermediates of both biosynthetic and lipolytic processes.

Hardon [14] first quantified the presence of MAGs (0.88%) and DAGs (5.55%) in HPO. More recently, Mozzon et al. [6] quantified the presence of 1(3)-P, 1(3)-O, and 1(3)-L (100–300 mg/100 g oil). The same authors identified the α,β- (1,2- + 2,3-racemic mixture) and 1,3-isomers of PP, PO, PS, PL, SO, OO, and OL. OO and PO, which globally account for 58%–66% of total DAGs, were the most represented. These data agreed with the representativeness of the different TAG species. Neither TAG biosynthesis nor enzymatic lipolysis involve 1,3-DAGs. Rather, they can originate from chemical (non-specific) hydrolysis of TAGs and/or isomerization of α,β-DAGs. Therefore, 1,3-DAGs are usually associated with undesired events, such as poor quality of raw matter and unsuitable management of oil extraction and storage [67].

**Figure 2:**
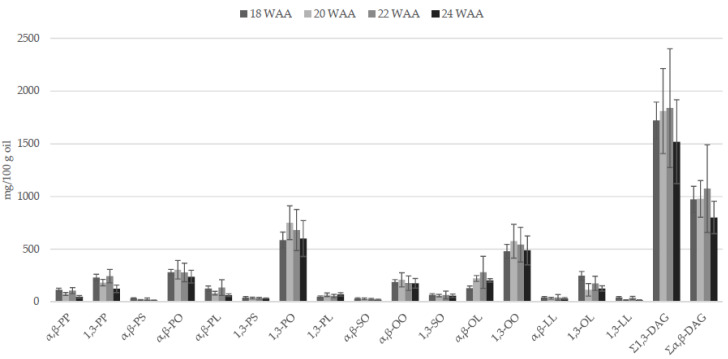
Diacylglycerol (DAG) contents (mg/100 g; mean ± SD; *n* = 3) in crude O × G hybrid palm oil during fruit ripening (data from [30]). P = C16:0; S = C18:0; O = C18:1; L = C18:2. WAA = week after anthesis.

The oil accumulation in fruit mesocarp of O × G hybrids (18–22 WAA) goes together with the biosynthesis of DAGs (Figure 2). An increase of OO and PO and a corresponding decrease of PP were also observed [30]. Since there were no differences in the ratio α, β-DAGs/1,3-DAGs during ripening, authors also concluded that the isomerization of α, β-DAGs produced by lipolytic enzymes could be the main cause of the presence of 1,3-DAGs.

### 2.3. Unsaponifiable Matter

Despite the interest for the recovering of health-promoting bioactive substances from crude vegetable oils (carotenoids, tocols), only a limited number of studies were carried out on the unsaponifiable matter (UM) of HPO. Literature data about non-glyceridic components of HPO are summarized in Table 4.

The amounts of carotenoids in crude HPO varies widely (1000–10,000 mg/Kg oil) [14,17,53,54,68]. Eleven different pigments were previously identified in HPO and crude oils of African and American parents: α-, β-, ζ- and γ-carotene, phytofluene, phytoene, α- and β-zeacarotene, neurosporene, lycopene. Literature data agree in identifying β-carotene (52%–60% of total carotenes) and α-carotene (33%–36% of total carotenes) as the most represented pigments of HPO. Lycopene accounts for 1%–8% of total carotenes in conventional PO, whereas in American and hybrid palm oils the lycopene contribution to total carotenoid content is less than 0.1% [54]. The almost colorless squalene (a diterpene hydrocarbon) ranges from 20 to 250 mg/Kg HPO [30,69].

Mozzon et al. [69] identified more than 40 alcoholic components (4-desmethylsterols, tocols, isoprenoid alcohols, triterpenols, and *n*-alkanols) in the UM of crude HPO. The most represented classes are 4-desmethylsterols and isoprenoid alcohols, which globally account for 79%–85% of total alcoholic substances of UM. An increase in the oil content of desmethylsterols, isoprenoid alcohols, tocols, and triterpenic alcohols was observed during fruit ripening, while *n*-alkanols and 4-methylsterols did not change significantly, as their progressive accumulation in HPO runs parallel to TAG synthesis [17,30].

About 97% of the phytosterol fraction, which globally accounts for 160–1400 mg/Kg of HPO [17,30,54,69], is made up of Δ5-sterols: β-sitosterol (58%–62% of total sterols), stigmasterol (13%–19%), and campesterol (12%–22%) are the most represented, whereas Δ7-campesterol is the only Δ7-sterol clearly identified in HPO. Cholesterol accounts for 2%–5% of total sterols. Literature data report a very similar composition of sterol fraction for *Eg* varieties, but lower relative percentages of β-sitosterol and stigmasterol and higher percentages of cholesterol in *Eo* oils [54,70,71]. The composition of the triterpenols, 4-methylsterols, and *n*-alkanols fractions does not differ significantly between the hybrid and African palm oils as well [30,69].

Triterpenic alcohols (4,4-dimethylsterols) globally account for 5%–6% of total alcoholic compounds (20–74 mg/kg oil). The two most represented components are cycloartenol (70%–75% of total triterpenols) and 24-methylenecycloartanol (14%–20%). Cyclobranol/cyclolaudenol and isoarborinol were also tentatively identified by Mozzon et al. [30,69].

The content of 4-methylsterols ranges between 7 and 15 mg/Kg g oil, corresponding to 1%–3% of alcoholic constituents of UM and to 0.1%–0.2% of total UM. Citrostadienol is the main 4-methylsterol in HPO (44.5%–50.3% of total 4-methylsterols), followed by obtusifoliol (14.3%–31.5%) and gramisterol (24.0%–35.4%) [69].

A series of *n*-alkanols with an even number of carbon atoms from 18 to 34 were identified in HPO, together with the odd carbon number alkanols C29, C31, and C33. The total amount of aliphatic alcohols ranges from 25 to 80 mg/kg. The relative distribution of *n*-alkanols follows a unimodal pattern, with an apex (maximum abundance) corresponding to the alcohol C32. Four isoprenoid alcohols with 20 carbon atoms and 1 to 4 double bonds were also identified. As previously stated, terpenols are the most represented class of alcoholic components of HPO unsaponifiable matter after 4-desmethylsterols [30,69].

The total amount of tocol chemical species (vitamin E) varies greatly in HPO, ranging between 10 and 2200 mg/kg Kg (3%–4% of total alcoholic constituents of UM). All eight α- (5,7,8-trimethyl-), β- (5,8-dimethyl-), γ- (7,8-dimethyl-), and δ- (8-methyl-) isomers of both tocopherols and tocotrienols have been identified, together with α-tocomonoenol. Tocotrienols are the most represented (γ-tocotrienol 40%–60% of total tocols; α-tocotrienol 15%–30%; δ-tocotrienol 5%–10%), while α-isomer is the most abundant tocopherol [17,30,53,54,68,69].

## 3. HPO as Food and Food Ingredient

### 3.1. Quality Parameters

The commercial value of the raw fatty substances is determined by different quality parameters, namely water, unsaponifiable and insoluble matter contents, and free acidity which provide an overall quantification of the non-glyceridic constituents.

Crude pressed HPO contains about 1% of unsaponifiable matter, 0.09%–0.20% of insoluble matter, and 0.20%–0.73% of water. The amount of free fatty acid (FFA) ranges between 0.35% and 2.91% [17,30,69], whereas higher free acidity levels (9.7%–36.7% as palmitic acid) were ascribed to the substantial delay between the removal of the mesocarp from the nuts and its thermal treatment, which allows intense lipolysis [16]. Interestingly, it has been observed that lipase activity in O × G fruits is strongly activated at low temperatures (+5 and −20 °C). This behavior is inherited from the African parent, while for *Eo* no significant lipase activity has been observed in the temperature range from −20 to +45 °C. Due to lower lipase activity, interspecific hybrids provide drupes with better stability after harvesting, as long as fruits are properly handled from harvesting to oil extraction [72].

In freshly pressed HPOs, no hydroperoxides were detected by conventional titrimetry, thus confirming their oxidative stability. However, a wide range of induction times (5.7–17.2 h) were measured in accelerated oxidation tests at 100 °C, likely due to different contents in antioxidant compounds (tocols and polyphenols) [6].

### 3.2. Food Uses

The Codex Committee on Fats and Oils [73] recently proposed the use of the term “Palm oil–high oleic acid (high oleic acid palm oil)” for oils derived from the fleshy mesocarp of hybrid palm fruit O × G (*Elaeis oleifera* × *Elaeis guineensis*). A set of physical (relative density, refractive index) and chemical (fatty acid composition, saponification value, iodine value, total unsaponifiable matter content, sterol composition, tocopherols and tocotrienols content) parameters were also provided for genuine crude oils. Particularly, oleic and linoleic acid percentages were set to 48.0%–60.0% and 9.0%–17.0%, respectively.

The utilization of HPO in the food industry is still very limited, due to the lack of information on functional and technological properties of HPO. In fact, previous studies on HPO have been focused on genetic, agronomic, or nutritional aspects and only a very limited number of researches have been conducted on the feasibility of replacing conventional PO and palm stearin with HPO and hybrid palm stearin (HPS). In fact, palm stearin is widely used to produce shortenings, margarines, and confectionery fats having a “zero” content trans-isomers of FAs. However, palm stearin is commonly blended and/or inter-esterified with PKO to improve organoleptic properties and retain the desired crystallization pattern. Flores Ruedas et al. [74] evaluated the physical, thermal, rheological, and microscopic properties of the HPS/PKO blends. Compared with conventional palm stearin/PKO, HPS/PKO blends showed similar consistencies, slightly lower melting and crystallization temperatures (about 2 °C), and a shorter crystallization time, thus promoting the HPS/PKO blends as a healthier alternative to conventional blends.

The processing of crude palm oil from the hybrid cultivars has also been scarcely studied. To avoid a large loss of neutral oil due to the high FFA level, crude palm oils usually undergo a physical deacidification at high temperatures (usually above 200 °C) and very low absolute pressures (below 5 mbar), during which residual carotenoids are thermally degraded. An integrated bleaching/degumming step precedes the deacidification, with the aim of removing phospholipids by precipitation and part of carotenoids via adsorption. The removal of carotenoids from crude palm oils is essential to lighten the color, thus guaranteeing their versatility and allowing their utilization in several food products (ice creams, breads, margarines). However, it was estimated that the amount of β-carotene lost during the refining process of palm oils would be enough to meet the worldwide need for vitamin A [75]. Hence, several technologies are currently being developed for carotenoids recovery before the physical or chemical refining of crude HPO. Vidoca et al. [76] assessed the adsorption onto polymeric resins, while Almeida et al. [77] modeled the kinetic of carotenes adsorption from crude HPO onto two commercial bleaching earths, an acid activated calcium bentonite and a neutral bentonite. The acid adsorbent showed better adsorption yields. Ribeiro et al. [78] found that the amount of β-carotene in HPO reduced considerably after bleaching by using both the acid and neutral bleaching earths, whereas the concentration of the α-isomer only showed considerable reduction after bleaching with the acid clarifying earth.

### 3.3. Nutritional Properties

A huge amount of literature has discussed and confirmed the involvement of dietary SFA, mainly palmitic acid, in the development of obesity, metabolic syndromes, type 2 diabetes, cardiovascular diseases, and some types of cancer [79]. Furthermore, in developed countries (Europe and North America) PO and PKO are used in their odorless and pale-yellow refined forms, while only a quarter of the palm oils worldwide are used as crude oil. Both volatile (off-odors, water) and non-volatile (free acids, unsaponifiable constituents, phospholipids) oil components are removed during the refining steps, thus causing both a decrease of nutritionally valuable components (polyphenols and tocols) [80] and the generation of new toxicants, such as chloropropanols (3-monochloropropane-1,2-diol, 3-MCPD; 2-monochloropropane-1,3-diol, 2-MCPD), glycidol, and their esters with the FAs. The deodorization step is the prime suspect in the generation of glycerol-based process contaminants, due to the strong conditions adopted, as previously reported. The expert panel on contaminants of the European Food Safety Authority (EFSA) first assessed the potential risk of 3-MCPD and glycidyl esters in 2016 and concluded that they are a concern for public health because of their genotoxic and carcinogenic activities. Particularly, the EFSA panel estimated that exposure to food 3-MCPD substantially exceeding the tolerable daily intake in younger age groups of the European population [81]. More recently, EFSA reviewed the risk assessment of 3-MCPD, after the Joint Food and Agriculture Organization (FAO)/World Health Organization (WHO) Expert Committee on Food Additives (JECFA) subsequently established a different safe level [82]. These drawbacks added to the open questions about the sustainability and ecological impact of palm plantations, thus reinforcing the bad reputation of palm oil among consumers.

The use of crude oils can avoid the exposure to process contaminants and significantly contribute to the dietary intake of substances with antioxidant properties (tocols, polyphenols). Among edible oils, extra virgin olive oil (EVOO) is certainly the most popular source of antioxidants and a great amount of literature supports the role of EVOO antioxidants in the prevention of chronic and degenerative diseases [83]. However, an excessive consumption of EVOO may still lead to adverse consequences [84]. Tocotrienols have recently gained increasing attention due to their higher biological effectiveness than tocopherols. Crude HPO brings together several interesting properties: a lower level of SFA than the traditional African palm oil together with a high amount of tocotrienols, carotenoids, and polyphenols. The latter ones have been recently studied by Rodríguez et al. [85], who detected the presence of several substances that have been already identified in EVOO (protocatechuic acid, protocatechuic aldehyde, vanillic acid, *p*-salicylic acid, syringaldehyde, syringic acid, ferulic acid) and measured a total amount of phenolic substances (190–260 mg gallic acid equivalent/kg oil) comparable to phenolic amounts in EVOO. Lucci and co-workers [86] found that the dietary crude HPO had a favorable effect on plasma lipids pattern related to cardiovascular risk factors and that this effect was not statistically different from that of EVOO. More recently, Ojeda et al. [87] explored the impact of daily consumption of crude oils (EVOO vs. HPO; 25 mL/day for 3 months) in adults aged 50–77 and found no significant differences on plasma antioxidant capacity and total phenolic content.

Due to these interesting findings, some authors suggested considering crude HPO as the “tropical equivalent of olive oil”. However, evidences to support this statement appear scarce. Spreafico et al. [88] assessed the effects of a high-fat diet containing conventional or hybrid palm oil on the lipid metabolism in a non-human primate model, the common marmoset. Results showed that animals fed with both oils developed non-alcoholic fatty liver disease (NAFLD) and animals fed an HPO diet demonstrated an even higher level of damage to the liver, which has been identified as an important risk factor for the development of cirrhosis and hepatocellular carcinoma. The same findings were confirmed by Sales et al. [89] in mice. These results suggest that the overconsumption of HPO through the most-consumed processed foods should be carefully monitored, as it could involve important alterations to hepatic metabolism. In addition, Gesteiro et al. [90] highlighted that no studies for assessing the nutritional effects of palm oils have been carried out in a Mediterranean diet context, where EVOO is the main culinary oil, thus avoiding a “direct match” between HPO and EVOO.

## 4. Conclusions

Conventional palm oil and its derivatives (stearins) are widely used in the food industry, due to their oxidative stability and technological properties. However, there is an increasing challenge for food technologists to develop healthier fats/oils with a reduced amount of saturated fatty acids, free from trans-fatty acids, and provide desirable functional and sensory properties.

The interspecific cross-breeding between the cultivated African palm and its wild South American relative produces oil palms that bring together several interesting properties like: better oil quality due to higher oleic acid percentage than conventional palm oil; longer productive life and shorter trunk than African palm; low lipolytic activity of fruit tissues; and resistance to the main diseases affecting palms. Hence, HPO could be a potential substitute for other monounsaturated fatty acid (MUFA)-rich vegetable oils, such as high oleic sunflower and safflower oils.

The Codex Committee on Fats and Oils recently provided HPO with the “dignity” of codified fat substance for human consumption and defined the physical and chemical parameters for genuine crude oils. However, the lack of information about the functional and technological properties of HPO is currently limiting its utilization in the food industry. To date, only a few studies have been conducted on the feasibility of replacing conventional palm oil and palm stearin with HPO and hybrid palm stearin. The processing of crude HPO has also been scarcely studied. The refining conditions should be revised to reduce the formation of undesirable process contaminants (chloropropanols, glycidol) and the whole process should be revised to accommodate technological solutions aimed to recover nutritionally valuable components (carotenoids, tocols).

To conclude, recent studies on the nutritional effects of HPO have softened the initial enthusiasm about the “tropical equivalent of olive oil”, suggesting that the overconsumption of HPO through most-consumed processed foods should be carefully monitored.

## Figures and Tables

**Table 1:** Fatty acid composition (% *w/w*) of mesocarp oil from interspecific hybrids.

C12:0 ^1^	C14:0	C16:0	C16:1 Δ9	C18:0	C18:1 ^2^	C18:2 Δ9,12	C18:3 Δ9,12,15	Reference ^3^
0.5–1.7	0.5–0.9	27.7–29.5	0.3–0.4	2.6–3.1	53.5–55.2	10.7–11.5	0.4–0.4	[6]
	0.9–0.9	37.0–43.5	0.2–0.2	4.0–4.3	38.7–43.4	10.7–12.7	0.3–0.4	[8]
	0.1–0.5	22.4–44.7		1.4–4.9	36.9–60.1	8.2–16.8		[9]
	0.1–0.6	22.3–34.3	0.2–0.8	1.5–3.1	48.2–61.4	10.5–15.1	0.4–0.7	[10]
0.0–0.1	0.5–0.9	27.3–32.5		3.4–6.1	48.0–52.5	11.3–11.8	0.4–1.3	[14]
tr.	0.4–0.9	29.3–35.5		3.0–4.6	50.2–53.4	10.3–13.9		[16]
		28.1–31.3		2.3–2.7	51.8–56.4	9.4–10.4		[17]
0.4–0.5	0.4–0.4	32.2–40.3	0.3–0.5	2.7–3.8	49.7–57.0	4.1–5.6	0.1–0.2	[30]
	0.2–0.3	26.2–32.5	0.1–1.4	1.5–4.8	48.4–58.2	9.9–13.0	0.3–0.5	[53]
	0.5–1,6	32.2–43.1		3.2–4.1	36.9–60.1	8.2–16.8		[54]
	0.4–0.8	36.2–41.4	tr.	0.4–1.5	48.2–53.3	6.5–9.3	0.1–0.7	[55] ^4^
	0.3–0.9	28.9–38.6		3.3–5.9	44.9–56.0	9.3–11.5		[56]

^1^ Fatty acid formula is: C (number of carbon atoms):(number of double bonds) Δx (position of double bonds). tr., traces. ^2^ C18:1 = sum of oleic and *cis*-vaccenic acids. ^3^ Notes: Sample origin; oil extraction system; number of samples. [6]: Colombia; pressure; 3. [8]: Costa Rica; solvent; 2. [9]: Malaysia; n.a.; 126. [10]: Malaysia; n.a.; 85. [14]: Congo, Malaysia, Colombia; n.a.; 3. [16]: Nigeria, Colombia; pressure; 7. [17]: Colombia; pressure; 21. [30]: Colombia; pressure; 12. [53]: Colombia; solvent; 3. [54]: Malaysia; n.a.; 3. [55]: Malaysia; n.a.; n.a. [56]: Nigeria; pressure; 14. ^4^. mol %.

**Table 2:** Fatty acid composition (% *w/w*) of mesocarp oil from interspecific hybrids.

C6:0 ^1^	C8:0	C10:0	C12:0	C14:0	C16:0	C18:0	C18:1 ^2^	C18:2 Δ9,12	Reference ^3^
	1.2–2.3	1.1–2.2	35.0–42.3	19.6–24.7	9.1–10.2	2.4–3.5	17.2–19.1	4.4.–4.7	[8]
0.2–0.2	3.2–3.4	2.7–2.9	44.4–46.8	18.1–18.6	7.9–8.8	2.1–2.2	14.8–16.3	3.2–3.4	[9,61]
0.2	4.0	3.5	50.0	16.5	7.8	2.2	13.1	2.4	[9,61]
tr.	1.3–3.2	1.8–3.2	40.6–49.0	17.4–22.1	8.0–9.5	1.5–2.5	14.1–18.5	1.0–4.5	[16]

^1^ Fatty acid formula is: C (number of carbon atoms):n (number of double bonds) Δx (position of double bonds). tr., traces. ^2^ C18:1 = sum of oleic and *cis*-vaccenic acids. ^3^ Notes: Sample origin; oil extraction system; number of samples. [8]: Costa Rica; solvent; 2. [9,61]: Malaysia; solvent; 12. [16]: Nigeria; solvent; 6.

**Table 3:** Triacylglycerol (TAG) composition (% *w/w*) of mesocarp and kernel oils from interspecific hybrid palms.

		Mesocarp Oil [Reference] ^3^					Kernel Oil [Reference] ^3^	
TAG m:n ^1^	TAG ABC ^2^	[6]	[9]	[15]	[17]	[30]	[9]	[61]
ΣC28							0.1	0.1–0.2
ΣC30							0.7	0.4–0.7
ΣC32							4.7	2.9–3.9
ΣC34							7.1	4.8–6.0
ΣC36							24.5	17.9–19.6
ΣC38							18.9	17.2–18.0
ΣC40							10.3	10.9–11.3
ΣC42							9.1	9.9–10.6
ΣC44							6.7	8.0–8.8
46:0	MPP	0.1 ± 0.0				0.1–0.5		
46:1	MMO + LaPO	0.3 ± 0.2				tr–0.3		
ΣC46			0.0–1.1				5.1	6.3–7.2
48:0	PPP	1.3 ± 1.3			0.0–0.6	1.5–2.8		
48:1	MOP	0.8 ± 0.0				0.4–0.6		
48:2	MLP	0.2 ± 0.1		0.4 ± 0.0	0.0 – 0.7	tr–0.1		
ΣC48			0.9–8.9				6.0	7.1–8.0
50:0	PPS	0.4 ± 0.3			0.0–0.2	0.2–0.6		
50:1	PPO	20.4 ± 0.2		17.0 ± 2.4	10.4–15.3	20.3–21.1		
50:2	PPL	5.5 ± 0.2		9.4 ± 0.9	5.6–9.4	2.5–3.2		
50:2	MOO	0.5 ± 0.1				0.3–0.5		
ΣC50			11.1–25.5				2.3	3.3–3.9
52:0	PSS	tr				tr		
52:1	POS	3.3 ± 0.2		2.8 ± 0.5	1.5–1.8	2.8–3.8		
52:2	PLS	1.6 ± 0.3				1.7–2.0		
52:2	POO	32.6 ± 2.4		23.4 ± 0.7	21.9–24.8	33.1–35.8		
52:3	PLO	11.2 ± 0.2		17.7 ± 0.8	17.8–20.2	7.4–8.9		
52:4	PLL + POLn	2.0 ± 0.0		6.7 ± 0.6	7.4–9.4	1.1–1.9		
ΣC52			43.5–50.5				1.9	2.9–3.3
54:1	SSO	0.3 ± 0.0			0.2–0.4	0.3–0.4		
54:2	SOO	2.6 ± 0.1		1.8 ± 0.3	1.1–2.5	2.3–3.5		
54:3	SLO	0.7 ± 0.7				1.4–1.5		
54:3	OOO	10.7 ± 0.2		7.6 ± 1.0	8.5–12.8	12.2–14.2		
54:4	OOL	4.7 ± 0.1		7.6 ± 0.6	8.5–11.3	4.6–5.2		
54:5	OLL	0.2 ± 0.1		3.2 ± 0.3	3.9–5.0	tr–0.3		
ΣC54			21.8–44.7				2.6	3.0–3.5
56:1	SOA	1.1 ± 0.1				0.9–1.1		
56:2	AOO	0.1 ± 0.0				0.1–0.1		
ΣC56			0.0–0.6					

^1^ TAG (triacylglycerol) formula is m (number of acyl carbons):n (number of double bonds). ^2^ ABC = fatty acid composition of TAG. Note that the abbreviations do not reflect the position of esterification of each FA. La = C12:0; M = C14:0; P = C16:0; S = C18:0; O = C18:1; L = C18:2; Ln = C18:3; A = C20:0. ^3^ Notes: sample origin; oil extraction system; number of samples. [6]: Colombia; pressure; 3. [9]: Malaysia; n.a.; 38. [15]: Colombia; pressure; 3. [17]: Colombia; pressure; 21. [30]: Colombia; pressure; 3. [61]: Malaysia; solvent; 12.

**Table 4:** Unsaponifiable matter constituents (mg/Kg oil unless % is indicated) of interspecific hybrid palm oil. Percentages refer to within class of unsaponifiable components.

Reference	[14]	[17]	[30]	[53]	[54]	[68]	[69]
**Carotenoids**	1070–1800	514–1375		1172.1–1449.6	800–2400		10,389.3 ± 1004.9
α-carotene				447.9–577.7	32.8–36.4		
β-carotene				724.2–911.8	51.6%–60.5%		
**Squalene**			20.3–83.1				247.4 ± 3.3
**4-desmethylsterols**							
Cholesterol			7.8–10.2 3.5%–5.4%		3%–5%		10.0 ± 2.6 1.8 ± 0.4%
Campesterol			18.8–47.6 11.8%–16.3%		20%–22%		93.1 ± 23.4 19.3 ± 1.2%
Ergosterol							11.0 ± 3.4 1.9 ± 0.3%
Stigmasterol			25.8–45.2 15.3%–16.3%		13%–19%		62.8 ± 10.8 13.1 ± 0.6%
Δ^7^-campesterol			2.3–3.6 0.8%–1.9%				1.7 ± 0.7 0.5 ± 0.3%
β-sitosterol			98.2–180.9 61.5%–62.4%		58%–61%		275.6 ± 57.4 59.3 ± 1.0%
Δ^5^-avenasterol			1.6–3.9 0.9%–1.4%				8.8 ± 1.3 1.9 ± 0.2%
Δ^5,24^-stigmastadienol			2.3–3.6 1.1%–1.9%				2.1 ± 1.4 0.5 ± 0.2%
Fucosterol							5.6 ± 2.9 1.1 ± 0.6%
Other unidentified sterols							2.1 ± 1.4 0.5 ± 0.2%
Total 4-desmethylsterols		469–1417	158.7–293.8		700–1400		472.7 ± 102.8
**Isoprenoid alcohols**							
Phytol			127.5–175.0				120.7 ± 26.1
3,7,11,15-tetramethyl-2,6-hexadien-1-ol							11.3 ± 2.1
3,7,11,15-tetramethyl-2,6,10-hexatrien-1-ol							7.7 ± 1.5
Geranylgeraniol			31.3–76.3				129.0 ± 31.7
Total Isoprenoid alcohols			160.7–251.3				269.3 ± 60.0
***n*** **-Alkanols**							
*n*-octadecanol							5.3 ± 2.1
*n*-docosanol			0.5–1.4				1.8 ± 1.3
*n*-tetracosanol			0.4–1.2				1.2 ± 0.5
*n*-hexacosanol			0.4–2.5				2.7 ± 0.2
*n*-octacosanol			3.0–5.2				7.3 ± 0.8
*n*-nonacosanol							tr
*n*-triacontanol			7.2–12.9				15.6 ± 1.6
*n*-hentriacontanol							0.7 ± 1.2
*n*-dotriacontanol			6.9–13.1				18.1 ± 6.5
*n*-tritriacontanol							0.7 ± 1.2
*n*-tetratriacontanol			2.2–37.4				8.2 ± 7.6
Total *n*-Alkanols			24.9–37.4				61.7 ± 17.0
**4-methylsterols**							
gramisterol							
obtusifoliol			2.7–5.2				
citrostadienol			4.2–9.8				
Total 4-methylsterols			6.9–14.9				12.7 ± 1.5
**4,4-dimethylsterols**							
Cycloartenol			14.6–24.9				
24-methylene-cycloartanol			2.0–3.4				
Isoarborinol			2.0–3.9				
9,19-cyclopropanesterol			0.8–1.6				
Total 4,4-dimethylsterols			20.0–33.7				74.0 ± 12.3
**Tocols**							
α-tocopherol			1.5–7.4	26.8–142.8	11–24%	4.2–5.1	27.1 ± 7.4 10.0 ± 0.2%
β- tocopherol						0–0.15	tr. 0.3 ± 0.3%
γ- tocopherol						0–0.27	tr. 0.3 ± 0.4%
δ- tocopherol						0–0.17	
α-tocotrienol				199.3–383.9	22%–31%	59.4–101.9	44.7 ± 13.7 15.0 ± 1.9%
β- tocotrienol						1.1–3.0	3.7 ± 1.2 1.4 ± 0.4%
γ- tocotrienol			9.4–18.9	666.0–998.4^2^	42%–51%	146.0–343.3	148.1 ± 23.3 59.7 ± 1.1%
δ- tocotrienol				41.2–45.6	5%–9%	9.4–17.9	31.8 ± 4.2 11.7 ± 0.8%
α-tocomonoenol						0.59–1.4	4.0 ± 1.7 1.6 ± 0.3%
Total Tocols (Vitamin E)		452–2189	10.9–26.2	937.6–1549.6	600–1000	222.0–471.9	259.3 ± 48.4

^1^ Notes: sample origin; oil extraction system; number of samples. [14]: Congo, Malaysia, Colombia; n.a.; 3. [17]: Colombia; pressure; 21. [30]: Colombia; pressure; 12. [53]: Colombia; solvent; 3. [54]: Malaysia; n.a.; 3. [68]: Costa Rica; pressure, solvent; 1. [69]: Colombia; pressure; 3. ^2^ β- + γ- isomers.
